# Cardiac Protection of Valsartan on Juvenile Rats with Heart Failure by Inhibiting Activity of CaMKII via Attenuating Phosphorylation

**DOI:** 10.1155/2017/4150158

**Published:** 2017-04-27

**Authors:** Yao Wu, Feifei Si, Xiaojuan Ji, Kunfeng Jiang, Sijie Song, Qijian Yi

**Affiliations:** ^1^Key Laboratory of Pediatrics in Chongqing, Chongqing 400014, China; ^2^Chongqing International Science and Technology Cooperation Center for Child Development and Disorders, Chongqing 400014, China; ^3^Department of Cardiovascular Medicine, Children's Hospital of Chongqing Medical University, Chongqing 400014, China

## Abstract

*Background*. This study was undertaken to determine relative contributions of phosphorylation and oxidation to the increased activity of calcium/calmodulin-stimulated protein kinase II (CaMKII) in juveniles with cardiac myocyte dysfunction due to increased pressure overload.* Methods*. Juvenile rats underwent abdominal aortic constriction to induce heart failure. Four weeks after surgery, rats were then randomly divided into two groups: one group given valsartan (HF + Val) and the other group given placebo (HF + PBO). Simultaneously, the sham-operated rats were randomly given valsartan (Sham + Val) or placebo (Sham + PBO). After 4 weeks of treatment, Western blot analysis was employed to quantify CaMKII and relative calcium handling proteins (RyR2 and PLN) in all groups.* Results*. The deteriorated cardiac function was reversed by valsartan treatment. In ventricular muscle cells of group HF + PBO, Thr287 phosphorylation of CaMKII and S2808 phosphorylation of RyR2 and PLN were increased and S16 phosphorylation of PLN was decreased compared to the other groups, while Met281 oxidation was not significantly elevated. In addition, these changes in the expression of calcium handling proteins were ameliorated by valsartan administration.* Conclusions*. The phosphorylation of Thr286 is associated with the early activation of CaMKII rather than the oxidation of Met281.

## 1. Introduction

With the increasing incidence of cardiovascular disease, the study of its pathogenesis and treatment has become a focus of medical research. Heart failure (HF) is a pathophysiological process involving systolic and/or diastolic cardiac dysfunction in which the heart cannot meet the metabolic needs of the body. Pediatric heart failure is primarily caused by congenital heart disease, myocarditis, cardiomyopathy, or severe respiratory disease [1, 2]. Although some drugs adopted for the treatment of heart failure in adults also show efficacy in children, not all of these pharmacological agents can be used in juveniles as the causes of heart failure in adult and pediatric patients are often distinct [3]. The development of therapies specifically targeted at children will help to improve the treatment of pediatric heart failure.

In myocardial cells, dysfunction of proteins associated with calcium homeostasis is thought to play a major causative role in cardiac dysfunction, electrophysiological instability, and arrhythmia [4]. Research has demonstrated that the activation of Ca^2+^/calmodulin-dependent protein kinase II (CaMKII) plays a key role in the development of HF [5]. CaMKII is a serine-threonine protein kinase with variety of biological functions [6]. There are four subtypes of CaMKII (*α*, *β*, *γ*, and *δ*) with differential distribution and function within particular tissues and cell types. CaMKII*δ* is the isoform primarily expressed in the heart, while a small amount of CaMKII*γ* can also be detected in cardiac tissue. Within cardiac myocytes CaMKII*δ*c is mainly localized in the cytoplasm, whereas CaMKII*δ*B and CaMKII*γ* are present in the nucleus [7, 8].

In cardiovascular disease, the overexpression of CaMKII leads to hyperphosphorylation of LTCC, ryanodine receptors (RyRs), and SERCA/PLN, which in turn contribute to cardiac excitation contraction coupling (ECC) and heart dysfunction [9]. CaMKII*δ*c transgenic rats demonstrate increased incidence of arrhythmia. This suggests that CaMKII regulates cardiac electrical activity, possibly by inducing sarcoplasmic reticulum (SR) calcium leakage through phosphorylated RyRs [10]. Animal experiments have shown that CaMKII*δ*-mediated increase in SR calcium leak through RyRs accompanies heart function decompensation, while the inhibition of CaMKII*δ* improved many symptoms associated with heart failure such as expansion of the heart chamber, pulmonary edema, myocardial fibrosis, and apoptosis [11].

In addition to phosphorylation-mediated activation sites, CaMKII may have other activation sites mediated by reactive oxygen species (ROS) [12]. In turn, CaMKII phosphorylates RyRs, but there is still controversy about the location of CaMKII phosphorylation sites on RyRs and whether the anchoring proteins are involved in this interaction. There is also interaction between CaMKII and cGMP-dependent protein kinase (PKG) on RyRs [13], but the role of CaMKII in the pathogenesis of juveniles HF is still far from clear.

Here, we investigate the mechanisms of CaMKII-mediated calcium regulation in myocardial cells. We also explore the effects of valsartan on heart failure and on the phosphorylation and oxidative activation of CaMKII in juvenile rats with cardiac dysfunction. Our findings inform future research on novel therapeutic approaches for the treatment of heart failure in pediatric patients.

## 2. Materials and Methods

### 2.1. Animals

Experiments were performed using male juvenile Sprague-Dawley (SD) rats aged from 21 to 28 days, with a body weight from 60 to 80 g. Animals were housed individually with free access to food and water and were maintained on a 12 : 12 h light-dark cycle with independent ventilation, temperature, and humidity controls. All animal studies were performed in accordance with the Guide for the Care and Use of Laboratory Animals of the National Institutes of Health, and all efforts were made to minimize suffering. The Ethics Committee of the Children's Hospital of Chongqing Medical University (permit number: SYXK2007-0016) approved all experiments. All animals (SPF grade) were purchased from the Animal Experiment Center of Chongqing Medical University.

### 2.2. Surgical Procedures

Naive rats were anesthetized with an intraperitoneal injection of 10% chloral hydrate (0.3 ml/100 g). Heart failure was induced by abdominal aortic constriction (AAC) according to a previously described method [14, 15] and rats were subsequently housed in our animal facilities. Briefly, through an abdominal incision, the intestine was pulled to the right side of the abdominal cavity and the posterior peritoneum was separated carefully to fully expose the abdominal aorta. The abdominal aorta, at 5 mm above the right renal vein, was carefully separated and was ligated with a parallel polished 23 G needle with the use of a polyester suture (4-0). The needle was extracted gently, resulting in 0.6 mm in diameter of abdominal aorta, and the incision was sutured. Sham-operated rats underwent a similar surgical procedure but without the abdominal aorta ligation.

### 2.3. Drug Preparation

Four weeks after the ACC procedure, valsartan (Tuoping, Tianda, China) solution was prepared fresh before intragastric administration by dissolving the drug in distilled water with carboxymethyl cellulose (CMC), resulting in a final CMC concentration of 0.5%. Every day for 4 weeks, valsartan (at a dose of 30 mg/kg body weight [16, 17]) or placebo (0.5% CMC in distilled water) was randomly administered to rats with HF in the HF + Val and the HF + PBO groups, respectively. During the same period, sham-operated rats were treated with valsartan or vehicle, as the Sham + Val group and the Sham + PBO group.

### 2.4. Doppler Echocardiogram Studies

Rats were anesthetized with 10% chloral hydrate and echocardiography was performed by ultrasound (GE, US) with a 12.5 MHz linear array ultrasound transducer. The left ventricle (LV) was assessed in both parasternal long-axis and short-axis views at a frame rate of 120 Hz. End-systole and end-diastole were defined as the phases in which the LV had the smallest and largest area, respectively. LV internal dimension systole (LVIDs), LV internal diastolic diameter (LVIDd), LV end-systolic volume (LVESV), LV end-diastolic volume (LVEDV), LV ejection fraction (LVEF), and LV fractional shortening (LVFS) were measured from the LV M-mode tracing with a sweep speed of 50 mm/s at the mid-papillary muscle level.

### 2.5. Histopathology

Freshly isolated rat hearts from all experimental groups were fixed in 4% paraformaldehyde for at least 24 hr. Heart tissues were then processed routinely for dehydration with 70–100% graded alcohol and embedded in blocks of paraffin wax. Serial sections of 4 *μ*m thickness were cut and mounted on silanized slides. Mounted sections were dried in an oven overnight at 60°C. Morphometric analysis of heart tissues from all experimental groups utilized hematoxylin and eosin (H&E) [18] staining, and collagen accumulation was assayed by Masson collagen staining [19]. Cross-sectional area of myocardial fibers and the proportion of collagen deposition were quantified using Image-Pro Plus 6.0 Analyzer. All sections from each experimental group were examined by the same researcher.

### 2.6. Western Blot Analysis

Whole protein was extracted from cardiac tissue (KeyGen Biotech, China) and quantified using a BCA assay (BioTeke Biotechnology, China). Total protein (30 *μ*g per lane) from the homogenate of isolated ventricular myocytes was separated by 10% SDS-PAGE (Beyotime Biotechnology, China) and transferred to a PDVF membrane. The PVDF membrane was blocked with T-TBS containing 5% BSA (Sigma, USA) at room temperature for 1 hour and then incubated with rabbit anti-phospho-CaMKII (1 : 1000 dilution, Cat.ab182647, Abcam, USA), rabbit anti-oxidized-CaMKII (1 : 1000 dilution, Cat. # 07-1387, Millipore, USA), rabbit anti-CaMKII (1 : 1000 dilution, Cat.ab181052, Abcam, USA), mouse anti-PLN (1 : 1000 dilution, Cat.ab2865, Abcam, USA), rabbit anti-phospho-PLN (1 : 1000 dilution, Cat.ab15000, Abcam, USA), rabbit anti-phospho-RyR2 (1 : 1000 dilution, Cat.LS-C358303, LSBio, USA), mouse anti-RyR2 (1 : 1000 dilution, Cat.ab2827, Abcam, USA), and mouse anti-GAPDH (1 : 5000 dilution, Cat.ARG10112, Arigo, Taiwan) antibodies overnight at 4°C. The membranes were then washed with Tris-buffered saline-Tween (0.05%) solution and incubated with HRP-conjugated secondary antibody (1 : 5000 dilution, Lianke, China) for 1 hour. The membranes were developed using a super ECL assay kit (KeyGen Biotech, China) and a G-BOX imaging system (Syngene, UK).

### 2.7. Statistical Analysis

All statistical analyses were performed with SPSS software (version 19.0). For normal distribution, comparisons of LV diameter, LV volume, heart function, left ventricular mass index (LVMI), and levels of phosphorylated CaMKII, oxidized CaMKII, CaMKII, phosphorylated RyR2, RyR2, phosphorylated PLN, and PLN among all the experimental groups were performed using one-way ANOVA, and the LSD method was applied to estimate pairwise comparisons. For nonnormal distribution, intergroup comparisons of cross-sectional area of myocardial fibers and collagen deposition were assessed with the use of the Kruskal-Wallis *H* test. The chi-square test was used for comparisons. For all statistical tests, significance was set at *P* < 0.05. Data are presented as the mean ± standard deviation, with the exception of the data not normally distributed which are shown as median (range).

## 3. Results

**Figure 1:**
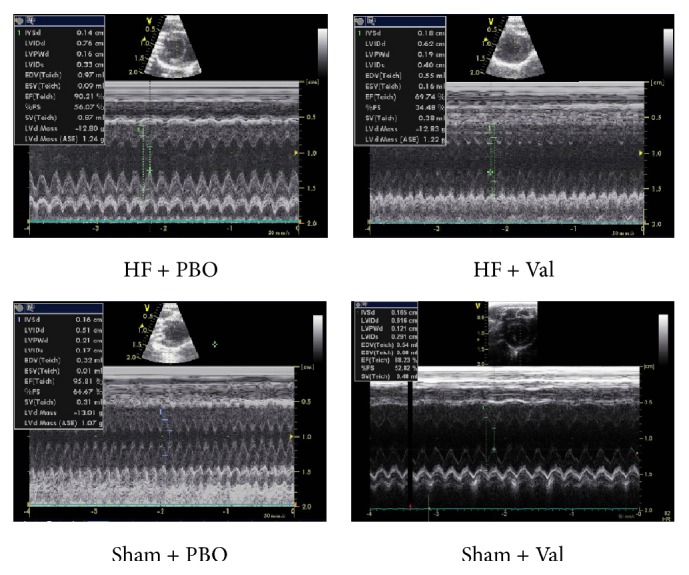
Representative M-mode images of transthoracic echocardiography in different groups.

To investigate the protective effects of valsartan, we evaluated cardiac function using a Doppler echocardiogram. As shown in Table 1 and Figure 1, treatment with valsartan prevented ventricular dysfunction due to AAC, as evidenced by improvements in LVIDs, LVESV, LVEF, and LVFS (all *P* ≤ 0.001); no significant changes were observed in the ACC-surgery rats treated with valsartan relative to the sham-operated, vehicle-treated control animals (*P* > 0.05). However, heart functions between Sham + PBO and Sham + Val are significantly different. LVIDs were increased (*P* = 0.044) with decreasing LVEF and LVFS (*P* = 0.002; *P* = 0.001) in the Sham + Val group compared to the Sham + PBO group.

**Figure 2:**
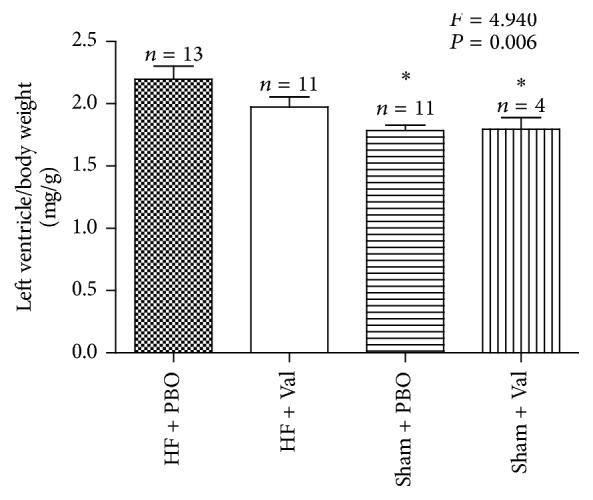
Effects of chronic increased pressure overload on left ventricle to body weight ratio in vehicle-treated (HF + BPO and Sham + BPO) or valsartan -treated (HF + Val and Sham + Val) rats. ^*∗*^*P* < 0.05 versus HF + PBO group.

As shown in Figure 2, the ratio of left ventricle to body weight was significantly increased in vehicle-treated heart failure rats indicating left ventricular hypertrophy, while this increase was much lower in the valsartan-treated rats with AAC (*P* = 0.006).

**Figure 3:**
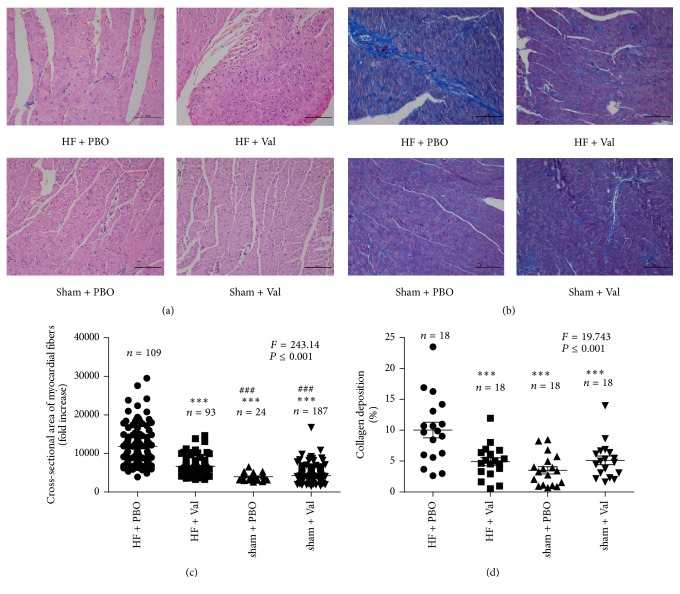
Representative myocardial H&E (a) staining and Masson collagen staining (b). Effects of chronic increased pressure overload on cross-sectional area of myocardial fibers (c) and collagen deposition (d) in valsartan-treated or vehicle-treated rats. ^*∗∗∗*^*P* ≤ 0.001 versus HF + PBO group. ^###^*P* ≤ 0.001 versus HF + Val group. Images were acquired at 200x magnification.

Our H&E staining further confirmed the inhibitory effect of valsartan on cardiac hypertrophy in AAC hearts (Figure 3). Valsartan lowered the cross-sectional area of myocardial fiber in the heart of AAC rat (*P* ≤ 0.001) (Figures 3(a) and 3(c)). Furthermore, valsartan also attenuated the increase in LV collagen volume induced by ACC, associated with cardiac hypertrophy (*P* ≤ 0.001) (Figures 3(b) and 3(d)).

**Figure 4:**
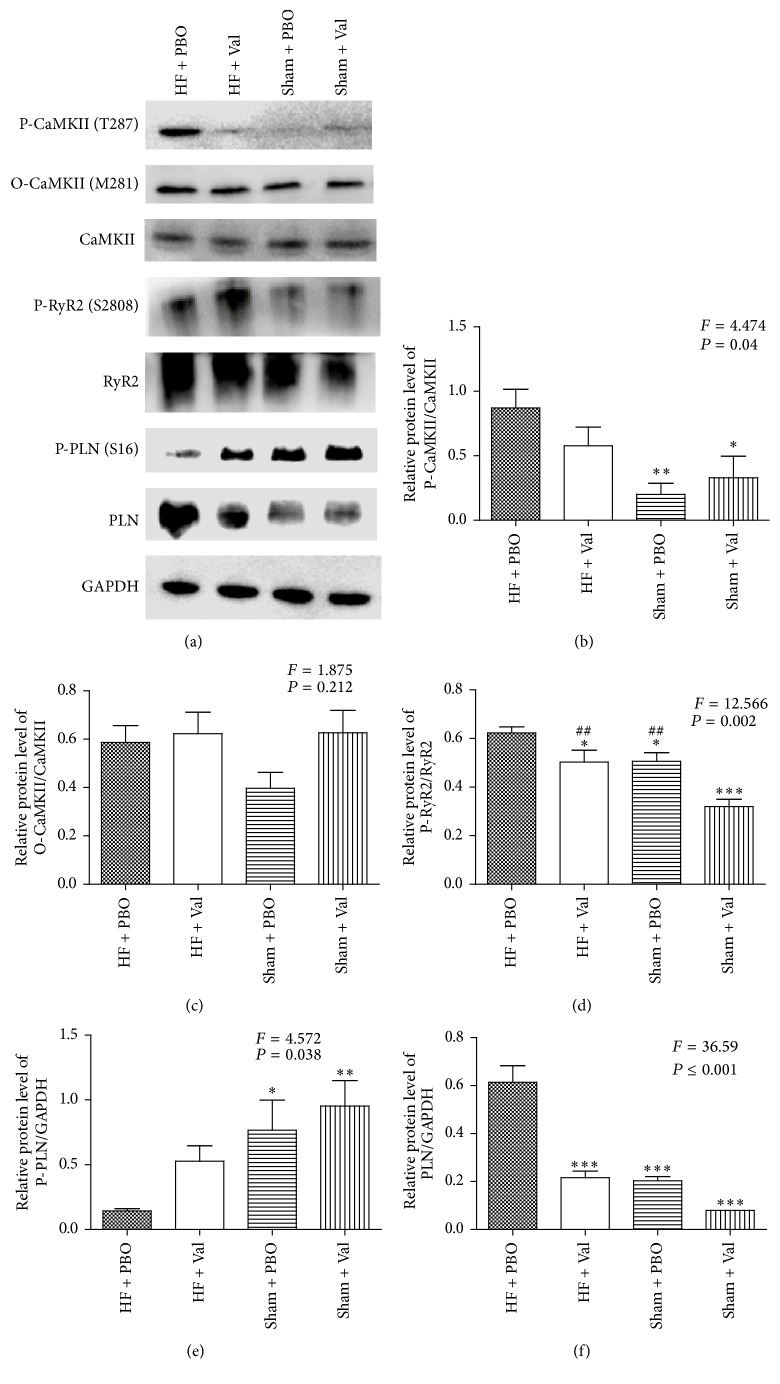
Relative CaMKII, RyR2, and PLN protein expression levels in isolated rat ventricular myocytes. Western blot analyses (a) and quantification of phospho-CaMKII (b), oxidized-CaMKII (c), phospho-RyR2 (d), phospho-PLN (e), and PLN (f) in juvenile rat ventricular cardiomyocytes (^*∗*^*P* < 0.05, ^*∗∗*^*P* < 0.01, and ^*∗∗∗*^*P* ≤ 0.001 versus HF + PBO; ^##^*P* < 0.01 versus Sham + Val; all *n* = 3).

We performed Western blot analysis to determine whether valsartan affected the levels of phosphorylated CaMKII and oxidized CaMKII following AAC. As shown in Figures 4(a) and 4(b), the levels of phospho-CaMKII were elevated approximately fourfold in heart tissue from vehicle-treated rats with AAC when compared with the sham-operated vehicle-treated controls. These elevated phospho-CaMKII levels were notably less in the valsartan-treated AAC rats relative to controls (*P* = 0.04). The expression of oxidized CaMKII, which represents reactive oxygen species- (ROS-) mediated CaMKII activation, did not differ among the four groups (*P* > 0.05) (Figures 4(a) and 4(c)). We also detected relative Ca^2+^ handling proteins in cardiomyocytes (Figures 4(a), 4(d), 4(e), and 4(f)). The levels of phospho-RyR2 (*P* = 0.002) (Figures 4(a) and 4(d)) and PLN (*P* < 0.001) (Figures 4(a) and 4(f)) were elevated and the level of phospho-PLN (*P* = 0.038) (Figures 4(a) and 4(e)) was decreased in heart tissue from vehicle-treated AAC rats when compared with the sham-operated vehicle-treated controls. These changes were less noticeable in the valsartan-treated AAC rats relative to controls.

## 4. Discussion

We found that AAC surgery, which resembles a cardiac dysfunction condition, significantly reduced the LV ejection fraction in juvenile rats. This is consistent with findings reported by others [20, 21]. Our results also showed that the AAC rats developed ventricular hypertrophy, shown by the increases in left ventricle to body weight ratio and changes in myocardial H&E and Masson staining. These results are in agreement with other studies [22]. In our study, the decreased cardiac function and ventricular hypertrophy in juvenile rats were improved by valsartan administration. These findings suggest that valsartan could be an effective therapeutic agent against cardiac dysfunction and cardiac hypertrophy caused by overload pressure. There is a possibility of application of valsartan to the treatment in children with heart failure. Interestingly, the sonography pictures show that the heart functions between HF + PBO and HF + Val are very similar. But the heart functions between Sham + PBO and Sham + Val are significantly different, indicating that valsartan may impair the normal heart function in healthy rats.

In the basal state, CaMKII is inactivated by the connection of the modulate region and the catalytic domain of CaMKII. In heart failure, increased CaMKII activity is initially through linking to Ca^2+^/CaM or the activation of phosphorylation and/or the oxidation sites of CaMKII. However, even if the concentration of Ca2+/CaM is very low in the basal state, there is still some small proportion of CaMKII proteins that are activated by autophosphorylation of Thr286/287 [23–25]. ROS-mediated activation of CaMKII has recently been described [26–29]. An earlier study found that, with the inhibition of calcium influx, CaMKII could be activated by ROS in Jurkat cells which had been pretreated with hydrogen peroxide [30]. Another study found that, in ventricular myocardium cells treated with hydrogen peroxide, the CaMKII-mediated phosphorylation of PLN was increased under oxidative stress conditions [8]. Met281/282 is the oxidative activation site of CaMKII, which performs similarly to activation by phosphorylation of Thr286/287, although these two modes of activation are independent [31].

Our study showed that the cardioprotective effect of valsartan is mediated by direct interruption of CaMKII signaling. Compared with sham-operated rats, phosphorylation of the Thr287 site of CaMKII was significantly increased in pups with overload pressure-induced cardiac dysfunction. However, there was no significant increase in the oxidation of Met281, suggesting that in early-stage heart dysfunction CaMKII is activated through phosphorylation rather than oxidation. Whether this is because of a low level of oxidative stress in rats with early-stage heart failure needs further research.

In addition, expression of RyR2 was remarkably increased in vehicle-treated AAC rats, contrasting that in vehicle sham rats. During the myocardial contractile period, a small amount of Ca^2+^ entering into cardiomyocytes triggers a large amount of Ca^2+^ released through RyR2 from the sarcoplasmic reticulum. During the diastolic period, most of the released Ca^2+^ was retaken by RyR2 from the cytoplasm. Dysfunctional RyR2 response to abnormal Ca^2+^ leak can deteriorate arrhythmia. The phosphorylation of PLN decreases in cardiac dysfunction in juveniles when compared with controls. Dephosphorylation of PLN induces active monomeric PLN, which can serve as an inhibitor of SERCA2a, causing low affinity of SERCA2a to Ca^2+^ and accelerating the progress of HF. In our study, valsartan also shows a protective role in maintaining calcium homeostasis through restoring the protein level of RyR2 and PLN.

In conclusion, valsartan shows a protective effect on the heart failure in juvenile rats maybe through the pathway of calcium handling in cardiomyocytes, especially by attenuating phosphorylation of CaMKII.

## Figures and Tables

**Table 1:** LV diameter, LV volume, and heart function in juvenile rats with or without valsartan administration $\left(\bar{x}\pm s\right)$.

Group	*n*	LVIDd (cm)	LVIDs (cm)	LVEDV (ml)	LVESV (ml)	LVEF (%)	LVFS (%)
HF + PBO	10	0.61 ± 0.12	0.39 ± 0.09	0.58 ± 0.33	0.16 ± 0.12	72.10 ± 5.02	36.50 ± 3.95
HF + Val	8	0.56 ± 0.13	0.22 ± 0.10^*∗∗∗*^	0.46 ± 0.27	0.04 ± 0.04^*∗∗*^	92.39 ± 5.51^*∗∗∗*#^	61.58 ± 10.48^*∗∗∗*#^
Sham + PBO	8	0.54 ± 0.09	0.20 ± 0.07^*∗∗∗*#^	0.41 ± 0.21	0.03 ± 0.03^*∗∗*^	93.94 ± 2.95^*∗∗∗*##^	63.37 ± 6.97^*∗∗∗*##^
Sham + Val	5	0.61 ± 0.04	0.32 ± 0.06	0.54 ± 0.08	0.09 ± 0.06^*∗*^	82.41 ± 10.48^*∗∗∗*^	45.4 ± 7.38^*∗∗*^
*F*		5.85	9.75	0.69	9.75	45.58	28.34
*P*		0.48	≤0.001	0.57	≤0.001	≤0.001	≤0.001

^*∗*^ *P* < 0.05, ^*∗∗*^*P* < 0.01, and ^*∗∗∗*^*P* ≤ 0.001 versus HF + PBO group; ^#^*P* < 0.05 and ^##^*P* < 0.01 versus Sham + Val group.
